# Association of Tumor Necrosis Factor-α -308G>A, -238G>A and -376G>A polymorphisms with recurrent pregnancy loss risk in the Greek population

**DOI:** 10.1186/s40738-021-00101-x

**Published:** 2021-04-10

**Authors:** Sofoklis Stavros, Despoina Mavrogianni, Myrto Papamentzelopoulou, Evaggelos Basamakis, Hend Khudeir, Alexandros Psarris, Peter Drakakis

**Affiliations:** 1st Department of Obstetrics and Gynecology ‘Alexandra’ General Hospital, Molecular Biology Unit, Division of Human Reproduction and Recurrent Abortions, National and Kapodistrian University of Athens, 80, Vasilissis Sofias Ave., 11528 Athens, Greece

**Keywords:** Tumor necrosis factor alpha, Variants, Recurrent pregnancy loss, Parity, Genotype, Allele frequency

## Abstract

**Background:**

Promoter region SNPs in TNF-α have been studied in association with Recurrent Pregnancy Loss (RPL) occurrence in various populations. Among them, −238G > A, −308G > A and − 376G > A have been frequently investigated for their potential role in recurrent abortions. The aim of the present study is to evaluate the correlation among TNF-α 238, TNF-α 308 and TNF-α 376 polymorphisms and recurrent pregnancy loss risk in Greek women.

**Methods:**

This study included 94 Caucasian women with at least two miscarriages of unexplained aetiology, before the 20th week of gestation. The control group consisted of 89 Caucasian women of proven fertility, with no history of pregnancy loss. DNA samples were subjected to PCR amplification using specific primers. Sanger sequencing was applied to investigate the presence of TNF-α 238, TNF-α 308, TNF-α 376 polymorphisms in all samples.

**Results:**

The TNF-α 238 and TNF-α 308 variants were both detected in RPL and control groups (7.45% vs 4.49 and 45.16% vs 36.73%, respectively), but with no statistically significant association (*p*-value 0.396 and 0.374, respectively). The TNF-α 376 variant was not detected at all in both control and RPL groups. When TNF-α 238 and TNF-α 308 genotypes were combined no association with RPL was detected (*p*-value = 0.694). In subgroup analysis by parity, RPL patients carrying the A allele reported less previous births.

**Conclusions:**

This is the first study demonstrating TNF-α 238 and TNF-α 308 gene expression and the absence of TNF-α 376 variant in Greek women with RPL. However, no association emerged between each polymorphism studied and the occurrence of recurrent pregnancy loss. Accordingly, TNF-α -308G > A, −238G > A and -376G > A variants are not considered genetic markers for identifying women at increased risk of recurrent pregnancy loss in the Greek population.

## Introduction

Recurrent pregnancy loss (RPL) is defined as three or more consecutive spontaneous abortions before the 20th week of gestation, as proposed by the European Society of Human Reproduction and Embryology, and occurs in approximately 1–2% of reproductively active couples [1–3]. RPL is recognized as a multifactorial disease with several well known causal triggering factors that include genetic defects, e.g., parental chromosome abnormalities, uterine anomalies, metabolic and endocrine disorders, e.g., hypothyroidism, luteal phase deficiency and poorly controlled diabetes, autoimmune abnormalities, i.e., antiphospholipid syndrome, and hereditary thrombophilia [4–7]. However, the etiology of RPL needs further elucidation, since nearly 50% of RPL cases are still classified as unexplained.

Emerging evidence indicates that endometrial immune dysregulation could be responsible for several, if not many, cases of RPL of unknown origin. Among the immune factors, cytokines seem to play a pivotal role in reproductive dysfunctions [8–11]. Tumor necrosis factor-alpha (TNF-α) is a key pro-inflammatory cytokine and plays an important role in apoptotic cell death and initiating an immune response. TNF-α is produced by macrophages and antigen-stimulated T-cells, yet other cells such as B-cells, neutrophils, and endothelial cells have been also described to produce TNF-α [12]. Interestingly, the circulating levels of TNF-α are higher in case of miscarriage compared to those reported in a successful pregnancy indicating that abnormal TNF-α levels adversely affect the progression of pregnancy [9, 13–15].

Several single nucleotide polymorphisms (SNPs) have been reported to regulate the transcription and production of TNF-α. In particular, promoter region SNPs in TNF-α have been studied in association with RPL occurrence. Among them, −376G > A (rs1800750), −308G > A (rs1800629) and − 238G > A (rs361525) have been frequently investigated for their potential role in recurrent abortions [16, 17]. Although in various populations the association between the aforementioned TNF-α variants and the risk of RPL has been examined, the results remain inconclusive [17–20]. Indicatively, in a recent meta-analysis TNF-α -308G > A polymorphism was shown to be strongly correlated with RPL occurrence in Asian populations rather than in European populations [21]. Regarding TNF-α -238G > A polymorphism, a strong association with RPL was observed in the Korean population [22], while TNF-α 238 and 308 variants were not significantly correlated with increased risk of recurrent miscarriage among Chinese women [23]. As for TNF-α -376G > A polymorphism, no association with recurrent miscarriages in Caucasian Italian women was revealed [24]. On the contrary, in Bahraini women TNF-α 376 variant was significantly associated with risk for recurrent abortions [18].

Given the controversial data upon TNF-α variants and RPL, the aim of the present study is to investigate the possible correlations between TNF-α 308, TNF-α 238, and TNF-α 376 polymorphisms and recurrent pregnancy loss risk separately and combined, thus revealing the genetic profile probably related to recurrent abortions occurrence in the Greek population. These three above-mentioned TNF-α variants were selected to be studied herein because they are among the most frequently reported TNF-α variants in association with RPL occurrence in various populations.

## Methods

### Study design

The study group included 94 Caucasian women with at least two miscarriages of unexplained aetiology, before the 20th week of gestation who visited the Recurrent Miscarriage Outpatient Clinic of Alexandra Hospital. Women with a history of thromboembolic, infectious, autoimmune, endocrine or chromosomal disorders, and cervical anatomical abnormalities were excluded from the study. The control group included 89 Caucasian women of proven fertility with no history of pregnancy loss. The study protocol was approved by the Ethics Committee of Alexandra Hospital and a signed informed consent was obtained from each participant of the present study.

### DNA isolation and sequencing

Peripheral blood (2-3 ml) was collected from all the recruited women. DNA isolation was performed using the PureLink Genomic DNA kit provided by Invitrogen Life Technologies. DNA samples were subjected to PCR amplification using specific primers for TNF-α 376, TNF-α 238, and TNF-α 308 polymorphisms. All primers were designed by TIB MolBiol and their sequences were as follows: for TNF-α -238G > A, forward primer: 5′-TTCCTGCATCCTGTCTGGAA, reverse primer: 5′-CAGCGGAAAACTTCCTTGGT-3′; for TNF-α -308G > A, forward primer: 5′-AGGCAATAGGTTTTGAGGGCCAT-3′, reverse primer: 5′-TCCTCCCTGCTCCGATTCCG-3′; for TNF-α -376G > A, forward primer: 5′-GCTGCACTCGATGTACCAC-3′, reverse primer: 5′-CAAGTTCTACAGAGCGAAGG-3′. The PCR protocol included adding 10X PCR Buffer minus Mg^2+^, 10 mM dNTP mixture, 1 μl 50 mM MgCl_2_, 1 μl Primer Sense mix, 1 μl Primer Antisense mix, 1 μl Template DNA, 0.3 μl Taq DNA polymerase and 17.2 μl of distilled water. The PCR conditions were 95 °C for 10 min and 95 °C for 1 min, 65 °C for 1 min, and 72 °C for 1 min for 29 cycles, with a final extension step at 72 °C for 10 min. The tubes were then incubated at 72 °C for 10 min. PCR products were subjected to agarose electrophoresis to verify for the expected length. Subsequently, Sanger sequencing was applied in an ABI PRISM 3130*xl* Genetic Analyzer (Applied Biosystems™) to determine the presence of TNF-α 238, TNF-α 308, TNF-α 376 polymorphisms in all samples. BigDye™ Terminator v3.1 Cycle Sequencing Kit (Applied Biosystems™) was used in the sequencing reactions.

### Statistical analysis

The differences between polymorphism frequencies were assessed by the chi-square test, while unpaired t-test was performed to evaluate the differences for age and body mass index (BMI) between groups. A *p*-value < 0.05 was considered as statistically significant. Statistical analysis was performed using STATA version 13.1 (STATA Corp. LP, College Station, Texas, USA).

## Results

### TNF-α variant presence in patients and controls

In our study three different polymorphisms of *TNF-α* gene were studied in 94 cases and 89 controls. In particular, the TNF-α -238G > A variant was detected in heterozygosity in 7 out of the 94 patients (7.45%; 7/94), while it was found to be present in 4 women of the control group (4.49%; 4/89). However, no statistically significant difference was observed between patient and control groups indicating that GA genotype can be equally detected either in women with RPL or controls (*p*-value = 0.396; Table 1). The AA genotype was only detected in 1 woman of the patient group (1.06%; 1/94) and in none in the control group (Table 1). Compared to controls, although there was no statistically significant difference, the A allele frequency was higher in RPL patient group (4.79% vs. 2.25%, respectively; *p*-value = 0.261; Table 1).

**Table 1:** Genotype and allele frequencies of TNF-α 238, 308 and 376 variants in RPL patient and control groups. Statistical significance was defined at the level of 5% (*p* < 0.05)

	**Total**	**RPL group**	**Control group**	**Patients versus Controls**	
	**(*N*** **= 183)**	**(*N*** **= 94)**	**(*N*** **= 89)**	**OR**	***p*-value**
**TNF-α − 238, n (%)**					
**GG**	171 (93.44)	86 (91.49)	85 (95.51)	1	
**GA**	11 (6.01)	7 (7.45)	4 (4.49)	1.73	0.396
**AA**	1 (0.55)	1 (1.06)	0 (0)		
**Allele, n (%)**				**X**^**2**^	***p*-value**
**G**	353 (96.45)	179 (95.21)	174 (97.75)	1.722	0.261
**A**	13 (3.55)	9 (4.79)	4 (2.25)		
	**Total**	**RPL group**	**Control group**	**Patients versus Controls**	
	**(*N*** **= 111)**	**(*N*** **= 62)**	**(*N*** **= 49)**	**OR**	***p*-value**
**TNF-α − 308, n (%)**					
**GG**	52 (46.85)	27 (43.55)	25 (51.02)	1	
**GA**	46 (41.44)	28 (45.16)	18 (36.73)	1.44	0.374
**AA**	13 (11.71)	7 (11.29)	6 (12.24)	1.08	0.901
**Allele, n (%)**				**X**^**2**^	***p*-value**
**G**	150 (67.57)	82 (66.13)	68 (69.39)	0.2653	0.607
**A**	72 (32.43)	42 (33.87)	30 (30.61)		
	**Total**	**RPL group**	**Control group**		
	**(*N*** **= 183)**	**(*N*** **= 94)**	**(*N*** **= 89)**		
**TNF-α − 376, n (%)**					
**GG**	183 (100.00)	94 (100.00)	89 (100.00)		
**Allele, n (%)**					
**G**	366 (100.00)	188 (100.00)	178 (100.00)		

In addition, TNF-α -308G > A variant was detected in heterozygosity both in RPL (45.16%; 28/62) and control groups (36.73%; 18/49), whereas no statistically significant association emerged between GA genotype and RPL patients (*p*-value = 0.374; Table 1). The AA genotype was present in 11.29% (7/62) and 12.24% (6/49) of the patient and control groups, respectively (*p*-value = 0.901; Table 1). Similarly, the A allele frequency was higher in RPL group, but with no statistically significant difference observed between groups (33.87% vs. 30.61%, respectively; *p*-value = 0.607; Table 1). Hence, TNF-α 238 and TNF-α 308 variants do not seem to correlate with the occurrence of recurrent pregnancy loss risk in Greek women. Additionally, when TNF-α 238 and TNF-α 308 variants were combined no association was detected between the two groups (p-value = 0.694; Table 2), although the A allele frequency was higher in RPL group (19.76% vs. 16.84%, respectively; *p*-value = 0.431; Table 2).

**Table 2:** Genotype and allele frequencies of TNF-α 238 and 308 variants in RPL patient and control groups. Statistical significance was defined at the level of 5% (*p* < 0.05)

	Total	RPL group	Control group	X^**2**^	***p***-value
	(***N*** = 111)	(***N*** = 62)	(***N*** = 49)		
**TNF-α − 238/308, n (%)**				3.749	0.694
**GG/GG**	50 (45.05)	25 (40.32)	25 (51.02)		
**GG/GA**	39 (35.14)	24 (38.71)	15 (30.61)		
**GG/AA**	12 (10.81)	6 (9.68)	6 (12.24)		
**GA/GG**	2 (1.80)	2 (3.23)	0 (0)		
**GA/GA**	7 (6.31)	4 (6.45)	3 (6.12)		
**GA/AA**	1 (0.90)	1 (1.61)	0 (0)		
**Allele, n (%)**				0.621	0.431
**G**	362 (81.53)	199 (80.24)	163 (83.16)		
**A**	82 (18.47)	49 (19.76)	33 (16.84)		

On the other hand, TNF-α -376G > A variant was absent both in RPL and control groups (Table 1) suggesting that it is not involved in the development of recurrent spontaneous abortions in Greek women. Therefore, the present study demonstrates the distribution of TNF-α 238 and TNF-α 308 SNPs in the Greek population and the absence of expression of TNF-α 376 variant.

### TNF-α variants and parity status

We further investigated possible associations between TNF-α 238 and TNF-α 308 genotypes, and parity status in RPL patients and controls. For TNF-α 238 variant, regarding possible association between GA genotype and parity status no statistically significant difference emerged between RPL patients and controls (33.33% vs 66.67%, respectively; *p*-value = 0.061, Table 3). Moreover, parity was less reported in RPL patients carrying the wild-type allele (GG genotype) compared to controls, reaching statistical significance (15.00% vs 85.00%, respectively; *p*-value < 0.001, Table 3). On the contrary, for TNF-α 308 variant, GA genotype was correlated with fewer cases of parity in RPL group compared to controls, a finding that reached statistical significance (28.00% vs 72.00%, respectively; *p*-value < 0.001, Table 3). Moreover, AA genotype presented a statistically significant association with the absence of parity in RPL group since none of the RPL patients with AA genotype reported previous births (*p*-value = 0.001, Table 3). As for the wild-type allele, parity was less reported in RPL patients with GG genotype compared to controls at a statistically significant level (13.79% vs 86.21%, respectively; *p*-value < 0.001, Table 3).

**Table 3:** Associations between TNF-α 238 and 308 variants and parity status. Demographic data for age and body mass index (BMI) are also included. Statistical significance was defined at the level of 5% (*p* < 0.05)

**TNF-α − 238**	**RPL group** **AA (*n*** **= 1)**	**Control group** **AA (*n*** **= 0)**		**RPL group** **GG (*n*** **= 86)**	**Control group** **GG (*n*** **= 85)**	***p*-value**	**RPL group** **GA (*n*** **= 7)**	**Control group** **GA (*n*** **= 4)**	***p*-value**
**Age (years), mean (sd)**	39.00 (−)	–		32.75 (5.16)	43.32 (14.64)	< 0.001	33.71 (4.86)	53.75 (23.29)	0.049
**Body mass index (kg/m**^**2**^**), mean (sd)**	27.01 (−)	–		25.03 (5.08)	25.37 (4.19)	0.654	22.34 (1.95)	28.69 (3.68)	0.004
**Parity, n (%)**						< 0.001			0.061
**No**	1 (100.00)	–		71 (100.00)	0 (0.00)		5 (100.00)	0 (0.00)	
**Yes**	0 (0.00)	–		15 (15.00)	85 (85.00)		2 (33.33)	4 (66.67)	
**TNF-α − 308**	**RPL group** **AA (*n*** **= 7)**	**Control group** **AA (*n*** **= 6)**	***p*-value**	**RPL group** **GG (*n*** **= 27)**	**Control group** **GG (*n*** **= 25)**	**p-value**	**RPL group** **GA (*n*** **= 28)**	**Control group** **GA (*n*** **= 18)**	***p*-value**
**Age (years), mean (sd)**	31.29 (4.54)	42.83 (14.69)	0.073	33.19 (4.88)	38.54 (10.58)	0.022	34.67 (4.39)	47.22 (15.75)	< 0.001
**Body mass index (kg/m**^**2**^**), mean (sd)**	24.18 (6.33)	25.10 (3.33)	0.774	24.12 (4.09)	24.85 (4.56)	0.563	23.80 (4.03)	27.24 (3.56)	0.006
**Parity, n (%)**			0.001			< 0.001			< 0.001
**No**	7 (100.00)	0 (0.00)		23 (100.00)	0 (0.00)		21 (100.00)	0 (0.00)	
**Yes**	0 (0.00)	6 (100.00)		4 (13.79)	25 (86.21)		7 (28.00)	18 (72.00)	

## Discussion

Our study demonstrates the presence of TNF-α 238 and TNF-α 308 polymorphisms in the Greek women population, while TNF-α 376 variant was absent. Notably, no significantly statistical association emerged between each polymorphism studied and the occurrence of recurrent pregnancy loss. Additionally, when TNF-α 238 and TNF-α 308 genotypes were combined no correlation with recurrent abortions was disclosed.

The association between TNF-α polymorphisms and RPLs has been investigated in many studies; however, the results remain contradictory and unreliable. Population diversity could explain such opposing conclusions. In particular, previous meta-analyses have shown that the risk of RPLs is significantly associated with the incidence of TNF-α −308G > A and − 238G > A polymorphisms in the overall population [25], while in another meta-analysis no association emerged between TNF-α −308G > A and RPL as investigated in Caucasian and Asian subjects [26]. On the other hand, a recent meta-analysis of 10 case-control studies in Asian and European populations provided diverse conclusions for *TNF-α* gene polymorphisms and RPL. In particular, TNF-α -308G > A polymorphism was found to be associated with an increased risk of developing RPL, whereas no statistical correlation was observed between TNF-α -238G > A polymorphism and RPL. The combined results of the studies revealed that women who are homozygous (GG) and heterozygous (GA) for TNF-α -308G > A polymorphism have an increased risk of developing RPL compared to women who carry the wild-type allele. In addition, when stratifying the results according to the geographical origin of the participants, Asian populations show a stronger association between TNF-α -308G > A polymorphism and RPL occurrence than European populations. In contrast, for TNF-α -238G > A polymorphism, no statistically significant difference was observed in the incidence of homozygous (AA), heterozygous (GA) and homozygous for the wild-type allele (GG) genotypes between women who have experienced recurrent pregnancy loss and fertile women [21].

In a prospective cohort study the frequency of TNF-α -308G > A variant was investigated in 1652 pregnant women with intrauterine fetal death, preeclampsia, preterm labor before 34 gestational weeks, and small-for-gestational-age (SGA) infants. The researchers concluded that there was no statistically significant difference in the incidence of TNF-α -308G > A between the group with the above-mentioned pregnancy complications and the control group [27]. Similar results have been revealed in a Chinese population by Liu and his colleagues [23], where it has been shown that TNF-α 308 did not correlate with women with a history of RPL and recurrent implantation failures (RIF) in IVF attempts. However, TNF-α 238 GG genotype was present more frequently in patients with unexplained recurrent spontaneous abortion (URSA), suggesting that it could be a risk factor in Chinese RSA patients. Consistent with our findings are the results of a recent case-control study where a clear lack of statistical significance was revealed between the genotype distribution of TNF-α -238G > A and -308G > A polymorphisms and the risk of recurrent miscarriages [28]. On the contrary, a patient-control study conducted in the Korean population showed that TNF-α -238G > A polymorphism could serve as a possible genetic risk factor for RPL [22].

Regarding TNF-α -376G > A polymorphism, Palmirrotta and his colleagues showed no association with recurrent miscarriages in Caucasian Italian women, but TNF-α -376G/−308A/−238G haplotype was associated with low TNF-α levels (*P* = 0.021) and miscarriage (*P* = 0.023) [24]. However, in another population, Bahraini women carrying the 376A genotype showed a statistically significant (*P* = 0.011) risk for recurrent miscarriages. Additionally, there were significant differences in the frequency distribution of -376G > A genotype (*P* = 0.002) between patients and control group [18]. All the observed differences in TNF-α gene polymorphism distribution could be attributed to genetic heterogeneity of the studied populations, since population-to-population differences are common in genetic association studies. Such discrepancies exist in different ethnic groups, and may affect the influence of the polymorphism on recurrent miscarriage risk. Moreover, differences in sample sizes, selection and exclusion criteria and specific study group recruitment could affect TNF-α allele frequencies and the resulting associations with RPL occurrence.

An interesting observation emerged upon association of TNF-α 238 and 308 polymorphisms and parity status in RPL patients, since parity was less reported in patients carrying the A allele. A similar disclosure has been published for susceptibility to pre-eclampsia, wherein an analysis by parity revealed that carriers of A allele and AA genotype frequency of maternal TNF-α -308G > A polymorphism were associated with an increased risk of pre-eclampsia [29].

Remarkably, elevated levels of TNF-α at the maternal-fetal interface induce activation of natural killer cells (NK cells) and subsequent damage to placental growth [30, 31]. Investigating a possible association between a *TNF-α* gene polymorphism spectrum and the levels of the respective protein and therefore its function, may elucidate the potential link to recurrent abortions. Additionally, the establishment of a panel of cytokine gene polymorphisms associated with recurrent abortions would serve as an important biomarker for the prediction of miscarriages, promoting the individualized treatment of women with a history of recurrent pregnancy loss, thus increasing successful pregnancy potential. It is a common observation that patients with the same diagnosis may respond differently to therapies and it is becoming increasingly evident that this differential responsiveness may be due to specific DNA variations at the genomic level. New technologies such as next-generation sequencing (NGS), and bioinformatics, will enable a better understanding of the role of various genetic polymorphisms that will facilitate the next breakthrough toward further improving the clinical success for each individual RPL patient. Consequently, the challenge for future medicine is to move from a population-based view to an individually-based one through the implementation of novel technologies in the daily medical practice, establishing the Precision Medicine in human reproduction [32, 33].

To our knowledge, the present work firstly reports the distribution of *TNF-α* gene polymorphisms in Greek women with recurrent pregnancy loss, while revealing lack of association between the above-mentioned variants and the development of RPL in the Greek population. Such novel findings could help to further elucidate the pathophysiology of RPL in the Greek population, highlighting the importance of genetic profiling of each RPL patient.

## Conclusions

In conclusion, the present study demonstrates TNF-α 238 and TNF-α 308 gene expression in the Greek women population and the absence of expression of TNF-α 376 variant. Moreover, it is disclosed that parity was less reported in RPL patients carrying the A allele. On the other hand, TNF-α 238 and TNF-α 308 variants were not correlated with the occurrence of recurrent pregnancy loss. Additionally, no association emerged between TNF-α 238 and TNF-α 308 genotype combination and recurrent abortions. Ultimately, TNF-α -308G > A, −238G > A and -376G > A variants are not considered genetic markers for identifying women at increased risk of recurrent pregnancy loss in the Greek population.

## Data Availability

All data generated or analysed during this study are included in this published article.

## Abbreviations

TNF-α: Tumor necrosis factor-alpha
RPL: Recurrent pregnancy loss
SNPs: Single nucleotide polymorphisms
BMI: Body mass index
URSA: Unexplained recurrent spontaneous abortion
NK cells: Natural killer cells
